# Case of Typhoid Fever, (Dothinenteritis) in Which the First Sound of the Heart Was Scarcely Audible—Death—Autopsy—with Remarks

**Published:** 1843-06-10

**Authors:** C. W. Pennock


### CASE OF TYPHOID FEVER, (DOTHINENTERITIS,)

*In which the first sound of the heart was scarcely audible—Death—Autopsy—With Remarks.*

By C. W. Pennock, M. D.

To the Editor of the Medical Examiner.

Sir,—During the summer of 1842, many of the cases of typhoid fever in the Philadelphia Hospital, Blockley, presented symptoms of unusual prostration, with very slight eruption of dark coloured spots, the colour of which resembled that of typhus, rather than that of dothinenteritis. The type of the fever in these cases, although not decidedly intermittent, approximated more to that form than usual in typhoid fever, and quinine was administered with great advantage. There was one peculiarity in the disease which very strongly arrested our attention; it was the modification of the systolic, or first sound of the heart. When heard, it was in every case extremely feeble; in many instances it was extinct. The annexed case, which was one of the first received in the wards, is selected, inasmuch as it proved fatal; and is especially interesting as exemplifying the pathological condition of the heart. The notes have been communicated to me by my friend Dr. H. SELDEN, who was Resident Physician at the Hospital at the time of the occurrence of the disease; and this communication is sent you, in the hope that it may direct attention specially to the physical signs presented by the central organ of the circulation in cases of dothinenteritis.

Yours, very truly,
C. W. PENNOCK.

—

Hugh Brown, æt. 28, entered the ward on the 23d of July, 1842. A large, muscular, well-made man. He is a native of Ireland, but has been employed for the past seven years in this country as a weaver. His general health was always good, with the exception of an attack of rheumatism, which occurred several years ago, and from which he recovered perfectly.

The present attack commenced on the first of this month, (July.) After having gone to bed as well as usual, he was seized with a chill, and pain in his limbs, which were followed by fever and sweating, with pain in breast—principally the præcordial region—palpitation and dyspnœa, nausea, with occasional vomiting, and pain in the back of the neck. In a day or two cough commenced, with serous expectoration. About this time there was slight delirium. Although the breathing became less difficult, the continuance of the other symptoms obliged him to enter the hospital. He has had no regular treatment, but took purges of senna and manna, which produced diarrhœa.

At the time of his entrance he had some fever, cough, and dyspnœa; pulse feeble, quick, and frequent. Dulness upon percussion over præcordial region, of not more than its usual extent; first sound of heart scarcely audible; second distinct, but not loud; impulsion feeble, and sounds distant.

No further note of the case was made till the 26th. In the mean time he had seven cut cups at one time, and six at another, applied over præcordia. Has been taking infusion of ipecac., and had a dose of oil. A blister has been applied over præcordia.

To-day, (July 26), feels about the same. No headach or other pain, except along the sternum when he coughs; slept badly; a little delirious during the night. Has had five stools during past twenty-four hours, thin, yellowish, and watery. Some stupor today and restlessness, with slight subsultus. Memory imperfect; slow in answering questions; hearing impaired; skin of a dusky tint, moist and pleasant temperature; perspiration on face considerable; countenance a little flushed; tongue red at tip, covered at root and base with a brownish fur; bitter taste in mouth; some nausea; conjunctiva slightly injected; abdomen somewhat tympanitic; no tenderness; neither sudamina nor spots. Percussion anteriorly on right side clear. Left, slight dulness from third ribs to nipple, elsewhere clear. Posteriorly moderate resonance, becomes dull towards inferior margin, more particularly on right side. Respiration loud anteriorly on both sides, and sonorous. Posteriorly feeble, particularly at lower portions of both lungs, where sonorous rhonchi, with occasional traces of mucous are heard.

*Heart.*—Impulsion not perceptible; both sounds feeble, first scarcely heard, (apparently a little roughened.)

Pulse small, feeble, but regular, 100.

Respiration high and irregular, 26.

℞. Hyd. Chlo. Mit. ½.

Ipecac. gr. ⅓.

Pulv. Opii, gr. 1-6th.

q. h. seconde.

Spt. Æth. Nit. ʒj.

q. h. seconde.

Other medicine discontinued.

27th. Slept well last night; no delirium; no cephalalgia; hearing about the same; skin of the body hot; that of extremities hot but moist; tongue as before, rather dry; great thirst; cough less frequent. Pulse feeble and quick, 104. Respiration not so high, irregular, about 28. Subsultus somewhat diminished.

*Heart.*—Impulse not felt; both sounds feeble; the first blowing, and scarcely heard, the second natural, save its feebleness. Percussion posteriorly, is flat on right side, from an inch above lower angle of scapula downwards, and anteriorly, as far as middle of axillary region. Respiration in this portion of lung is very feeble and bronchial. In left lung, respiration, though feeble, is stronger than that of right.

Four cut cups to lower part of right lung posteriorly.

Medicine continued.

28th. Intelligence about the same. Had some delirium during night, but slept tolerably well; hearing not improved; no cephalalgia; flush of face of duller colour; skin less warm; perspiration considerable; tongue dry, brown and red at edges. Pulse 116, small and feeble; subsultus diminished; a spasmodic action of lower jaw very frequent. Respiration imperfect, 36, with very little expansion of chest in the act; less cough; very little sputa. Abdomen supple, but seems to contain a good deal of gas. Had three stools during night.

Percussion less dull on right side posteriorly than yesterday; and respiration more vesicular, and louder.

Heart as yesterday.

Medicine continued.

29th. Slept very little; muttering delirium during night; more delirium this morning than was observed yesterday; subsultus diminished, but twitching about mouth very frequent; flush of face increased; much heat about forehead; heat of body less; perspiration considerable; sudamina in considerable number about neck and abdomen; conjunctiva injected; neither dilatation nor contraction of pupils; tongue moist, cleaning at tip; slight ptyalism. Pulse rather fuller, still very feeble, 120. Respiration irregular, 40. Some abdominal tenderness. Stools of good colour, but thin; water has not passed freely, (eight oz. of a reddish colour drawn off;) abdomen tympanitic.

No change in physical signs.

Medicine discontinued.

30th. The stimulants administered yesterday producing little effect they were discontinued, and eight grains of carb. of ammonia, and a drachm of Hoffman's anodyne were substituted. This to be given every two hours. A blister was applied to back of neck, which he tore off before it produced any effect upon the surface; was delirious through night, and slept none. To-day there is slight delirium, but great stupor; difficulty of speech; inability to protrude tongue; subsultus increased; face flushed as before; respiration slower, more expansive, but more laboured and irregular; some tympanitis; a slight eruption, resembling that of typhus, observed on abdomen, principally confined to its left side; skin warm on body and extremities; considerable degree of perspiration on face and arms; tongue commencing to clean; no sordes on teeth; pulse more feeble, 140; conjunctival injection less; pupil changes rapidly from dilatation to contraction.

Ordered ten grains of carb. of ammonia, with a drachm of Hoffman's anodyne, to be given every alternate two hours, with ten minims of oil of turpentine, and three of oil of amber.

Evening.—About 6½ o'clock had an *immense* discharge from his bowels of thin, dark, bloody, fœtid matter, after which he became much more feeble. Perspiration over whole body. Stimulants were administered in large doses, sinapisms applied to abdomen and extremities, and hot spirits poured over body.

These measures failed to produce any reaction, and the patient died at 10½ o'clock, P. M.

*Sectio-Cadaveris seventeen hours after death.*

Blood perfectly fluid, and ran from the nose in large quantities when the body was moved.

*Lungs* congested; soft at lower part posteriorly, principally on right lung; no induration of any portion of them.

*Heart.*—No effusion into pericardium, or other evidence of pericarditis; muscular structure of the organ of a pale red colour, softened and flabby; lining membrane softened, so as to be easily separated; in the muscular structure of the columnæ carneæ are numerous yellow points, which give a fawn colour to those portions; the whole internal parietes much stained by imbibition.

*Stomach.* — Mucous membrane slightly injected near the pylorus, and at posterior side; general softening of the whole mucous membrane.

*Liver* very pale; soft in right portion; gives a crackling sound under the knife. Left lobe not so pale or soft, and does not crackle under the knife; none of it congested; in the thickest portions there are globules of air, possibly from commencing putrefaction.

*Spleen* large, very soft, and congested.

*Kidneys.*—External surface mottled, and of a yellowish white colour; internally deep red, extending throughout cortical substance. Tubular portion natural. Left kidney much enlarged.

*Mesenteric glands* much enlarged.

*Duodenum* perfectly healthy in appearance, with some viscous congestion.

*Jegunium* healthy, and no enlargement of glands corresponding to those parts.

About two feet and a half above the illeo-colic valve the first of the *glands of Peyer* are found inflamed and ulcerated; they are much more so in proceeding downwards towards the cœcum. One gland, a few inches above the valve, is ulcerated through to the peritoneum, and is completely gangrenous. Ulceration is observed immediately at the valve.

Colon highly congested, and stained—of a blackish red colour; no ulceration observed; contains a great quantity of fluid blood of a very black colour.

Brain not examined.

*Remarks.*

The medical profession is indebted to Dr. Stokes, of Dublin, for first directing attention to the peculiar modification of the cardiac systolic sound in certain forms of typhoid fever. This very important feature in the pathological symptoms of the disease, does not seem to have claimed much attention from the American physicians; at least, the writer is not aware of the existence of any cases of typhoid fever published by American practitioners, in which the peculiar cardiac symptom has been recorded.

In the treatment of this form of the disease, Dr. Stokes, with great reason, insists upon the expediency of the liberal use of wine and of tonics; a plan of practice which, in the cases in the Philadelphia Hospital, was attended with the happiest results. In some instances, where the first cardiac sound was entirely extinct, and the pulsations of the heart scarcely perceptible, under the influence of wine, tonics, and nourishing diet, the force of the central organ of the circulation increased, and with that its first sound was restored.

The case just reported is also interesting, as exemplifying the peculiar fluid and dissolved state of the blood, which is often seen in fevers of a low type.

